# Risk perception and spatial mobility on the Camino de Santiago: travel patterns analysis and the choice of Jacobean routes

**DOI:** 10.3389/fpsyg.2026.1899526

**Published:** 2026-08-10

**Authors:** Mar Durán, César San-Juan, Eulogio Real, Lucrezia Lopez

**Affiliations:** 1 Faculty of Psychology, University of Santiago de Compostela, Santiago de Compostela, Spain; 2 Faculty of Psychology, University of the Basque Country (UPV/EHU), Donostia-San Sebastián, Spain; 3 Faculty of Geography and History, University of Santiago de Compostela, Santiago de Compostela, Spain

**Keywords:** fear of crime, gender differences, natural environments, perceived safety, spatial behaviour

## Abstract

This study examines how fear of crime and perceived risk influence spatial mobility strategies in natural environments. Drawing on environmental criminology and fear-of-crime research, the article analyses how sociodemographic characteristics, prior knowledge of crime, and perceptions of safety shape route choices and walking behaviour among pilgrims on the Camino de Santiago. The study is based on a survey of 1,080 pilgrims who completed an online questionnaire about perceived safety, knowledge of crime, and preferences for different types of routes. Associations between variables were analysed using contingency coefficients and multiple correspondence analysis to identify underlying behavioural patterns. The results reveal clear differences in spatial behaviour linked to gender, age, and perceived vulnerability. Women and younger pilgrims show a stronger preference for walking accompanied and for routes with higher social presence, while men and older participants are more likely to select solitary or highly natural routes and to walk alone. Perceptions of risk and prior knowledge of crime also influence these mobility strategies. The findings highlight the importance of subjective perceptions of safety in shaping the use of natural environments and extend fear-of-crime research beyond urban settings. The study contributes to environmental criminology by showing how perceived risk structures spatial behaviour in large-scale natural routes, with implications for the design, management and safety of recreational and pilgrimage pathways.

## 1 Introduction

Fear of crime has long been recognised as a central concern within criminological research, shaping how individuals perceive, interpret and use public space. Beyond the objective probability of victimisation, subjective perceptions of risk influence everyday spatial behaviour, including route choices, mobility strategies and the avoidance of certain environments (Ferraro, 1995; Hale, 1996; Jackson, 2005). Individuals frequently adjust their daily practices in accordance with their perceived vulnerability, adopting precautionary behaviours such as avoiding isolated areas, travelling in company, or selecting routes on which the presence of other people enhances their sense of safety. The present study applies this perspective to a setting that has received little attention in the fear-of-crime literature, namely a large-scale natural and cultural route, in order to examine how perceived risk shapes the spatial mobility of pilgrims on the Camino de Santiago.

Within this perspective, environmental criminology has emphasised the importance of spatial and situational factors in shaping both the opportunities for crime and the perception of risk. The physical and social characteristics of an environment—such as visibility, accessibility, surveillance and the presence of other users—play a key role in structuring how individuals assess safety and move through space (Brantingham and Brantingham, 1991; Clarke, 1997; Felson, 2002). Research in this field has traditionally concentrated on urban settings, including streets, neighbourhoods and public parks. More recently, however, studies have demonstrated that environmental design and spatial configuration can substantially influence perceptions of safety across a wide range of public environments (Cozens and Love, 2015; Kuen et al., 2022; Wen et al., 2024). For clarity, the present article uses these terms as follows: urban settings denote densely built and populated spaces such as streets, neighbourhoods and squares; public environments refer to any openly accessible space, whether built or green, in which users encounter unknown others; and natural environments denote large-scale, low-density landscapes—forests, trails and pilgrimage paths—characterised by limited surveillance, vegetation cover and remoteness from urban centres.

Despite this extensive body of work, considerably less attention has been devoted to the perception of crime risk in large-scale natural environments. Recreational landscapes such as hiking routes, forest trails and pilgrimage paths combine the characteristics of leisure spaces with environmental features that may heighten perceived vulnerability, including isolation, low population density and limited surveillance. Such features may give rise to ambivalent perceptions among users: although natural environments are frequently associated with psychological restoration and well-being (Kaplan and Kaplan, 1989), they may also evoke uncertainty or insecurity owing to their relative remoteness and unfamiliarity. The perception of safety in this context therefore entails not only the absence of crime or fear of crime (Durán and Pasca, 2025) but also a sense of control over the natural environment. Although studies such as those of Groeneveld et al. (2025) and Matias et al. (2022) underline the value of contact with nature and of physical activity on nature trails, the realisation of these benefits depends on the experience being perceived as safe and free from threat. This perception of safety, beyond its social and personal determinants, is also strongly linked to the characteristics of the physical environment, including its design, order, lighting, spatial structure and maintenance (Qin et al., 2025; Park et al., 2025). While such research has been conducted predominantly in urban areas, some evidence also addresses semi-urban routes. Wang et al. (2022), for example, examined safety perceptions among tourists in urban forests in China and found that visual quality, the completeness of facilities and accessibility were significant predictors of perceived safety. A forest possesses an aesthetic value distinct from that of a park, and forest bathing, as a form of natural therapy, has been shown to promote physical and mental health to a far greater extent than a park (Farrow and Washburn, 2019). Nevertheless, because forests constitute wilder natural areas, they are often regarded as more dangerous; consequently, some tourists restrict their options and relatively few choose forest environments, particularly for outdoor activities involving children (Sonti et al., 2020). It is therefore especially important to alleviate the concerns and anxieties of users—in this case, pilgrims—taking into account, moreover, the natural environments, distant from urban centres, through which the various Jacobean routes pass.

Taken together, these observations call for an explicitly psychological framework capable of linking environmental perception, emotion and behaviour. Three complementary perspectives inform the present study. First, Attention Restoration Theory (Kaplan and Kaplan, 1989) holds that natural settings can restore cognitive and emotional resources, but only when they are experienced as compatible with the individual’s purposes and free from threat; perceived danger undermines the sense of “being away” and the effortless fascination on which restoration depends. Second, prospect–refuge theory (Appleton, 1975; Fisher and Nasar, 1992) explains how the spatial configuration of a setting—the balance between open visibility (prospect), places of concealment (refuge) and the possibility of escape—shapes feelings of safety or exposure, a mechanism that is especially salient on isolated trails with dense vegetation and few escape routes. Third, the risk-interpretation model (Ferraro, 1995) conceptualises fear of crime as the outcome of a subjective appraisal in which perceived risk is weighed against the individual’s sense of control and coping capacity, so that the same environmental cue may heighten fear in one person yet not in another. Together, these frameworks position perceived safety as a psychological appraisal—of restoration, exposure and control—that stands between the objective features of an environment, sociodemographic vulnerability and the spatial strategies that individuals adopt. They also clarify the psychological variables examined here: perceived safety and perceived vulnerability (appraisal), prior knowledge of crime (a cognitive input to that appraisal) and route and companionship preferences (the behavioural outcomes).

Preferences for a given type of route (natural, urban, busy or solitary) are directly related to perceptions of safety and to sociodemographic characteristics. Evidence indicates that women report higher levels of fear of crime or harassment and a greater reliance on environmental cues in order to feel safe (Durán and Pasca, 2025; Rišová and Sládeková Madajová, 2020), which translates into a preference for busy routes and for walking in company. By contrast, men and older individuals tend to perceive lower levels of vulnerability, to select more solitary or natural routes, and to be more willing to walk alone (Zhang et al., 2021). These demographic differences in the experience of space reinforce the need for models that incorporate gender and age as moderators of perceived safety.

The Camino de Santiago, or Way of St James, is a network of Christian pilgrimage routes that since the Middle Ages have converged on the shrine of the apostle St James in Santiago de Compostela, in north-western Spain. The Jacobean routes are not a single path but a system of waymarked itineraries—among them the French Way, the Northern Way, the Portuguese Way, the Primitive Way and the Vía de la Plata—that cross the Iberian Peninsula and continue into France and other European countries. They traverse markedly heterogeneous environments, from dense urban cores to long stretches of forest, farmland and sparsely populated mountain terrain, and are travelled on foot or by bicycle over journeys that typically last from several days to several weeks. This combination of cultural and spiritual significance, an international and socially diverse body of users, and prolonged exposure to low-density natural settings makes the Camino an unusually informative case for studying perceived risk. It functions as a real-world setting in which fear-of-crime mechanisms can be observed beyond the urban micro-settings to which they are usually confined; and because the conditions that drive perceived vulnerability here—isolation, limited surveillance and reliance on fellow travellers—are common to long-distance trails elsewhere, the patterns it reveals are likely to extend to other recreational and pilgrimage routes well beyond Spain.

The Camino de Santiago constitutes a particularly relevant context in which to examine these dynamics. As an extensive network of pilgrimage routes traversing rural landscapes, forests, small towns and, occasionally, urban areas, the Camino combines cultural, spiritual and recreational motivations with prolonged exposure to diverse environments. Pilgrims frequently travel long distances on foot or by bicycle, taking daily decisions regarding routes, companionship and travel strategies. In such circumstances, perceptions of safety and of the risk of victimisation may play an important role in shaping spatial mobility patterns, including preferences for solitary or busy routes and the decision to travel alone or accompanied.

Previous research has demonstrated that perceptions of safety in public spaces are not determined solely by objective crime levels but are also shaped by individual characteristics and social factors. Sociodemographic variables such as gender and age have consistently been associated with differences in perceived vulnerability and fear of crime (Pain, 2001; Stanko, 1990). Women and younger individuals, for instance, tend to report higher levels of perceived risk in public environments and are more likely to adopt precautionary spatial strategies. Moreover, prior knowledge of crime—whether acquired through personal experience, media reports or informal communication—can shape individuals’ expectations of risk and influence their spatial decisions (Cozens and Love, 2015; Focás, 2015).

These dynamics are particularly salient in settings such as pilgrimage routes, where mobility unfolds across long distances and heterogeneous environments. In such contexts, individuals continuously evaluate environmental cues and adjust their behaviour accordingly, balancing motivations related to solitude, nature and personal reflection against concerns about safety and vulnerability. Understanding how these perceptions influence spatial behaviour is therefore essential both for analysing the social use of natural environments and for informing policies aimed at improving safety and accessibility along recreational routes.

In light of the foregoing, the present study examines how the perceived risk of victimisation, prior knowledge of crime and sociodemographic characteristics shape the spatial mobility strategies of pilgrims on the Camino de Santiago. Drawing on environmental criminology and fear-of-crime research, the study pursues three specific objectives: first, to determine whether gender and age are associated with differences in route preferences and in the decision to walk alone or accompanied; second, to analyse how perceived safety and prior knowledge of crime relate to these mobility strategies; and third, to identify the underlying behavioural patterns that structure pilgrims’ use of these natural and culturally significant environments. Accordingly, three research questions are addressed: (RQ1) To what extent do sociodemographic characteristics, in particular gender and age, shape pilgrims’ route preferences and their willingness to walk alone or in company? (RQ2) How do perceived safety and prior knowledge of crime relate to these spatial mobility strategies? (RQ3) Which underlying dimensions account for the relationships among the sociodemographic, perceptual and environmental variables involved in pilgrims’ spatial behaviour?

**Figure 1:**
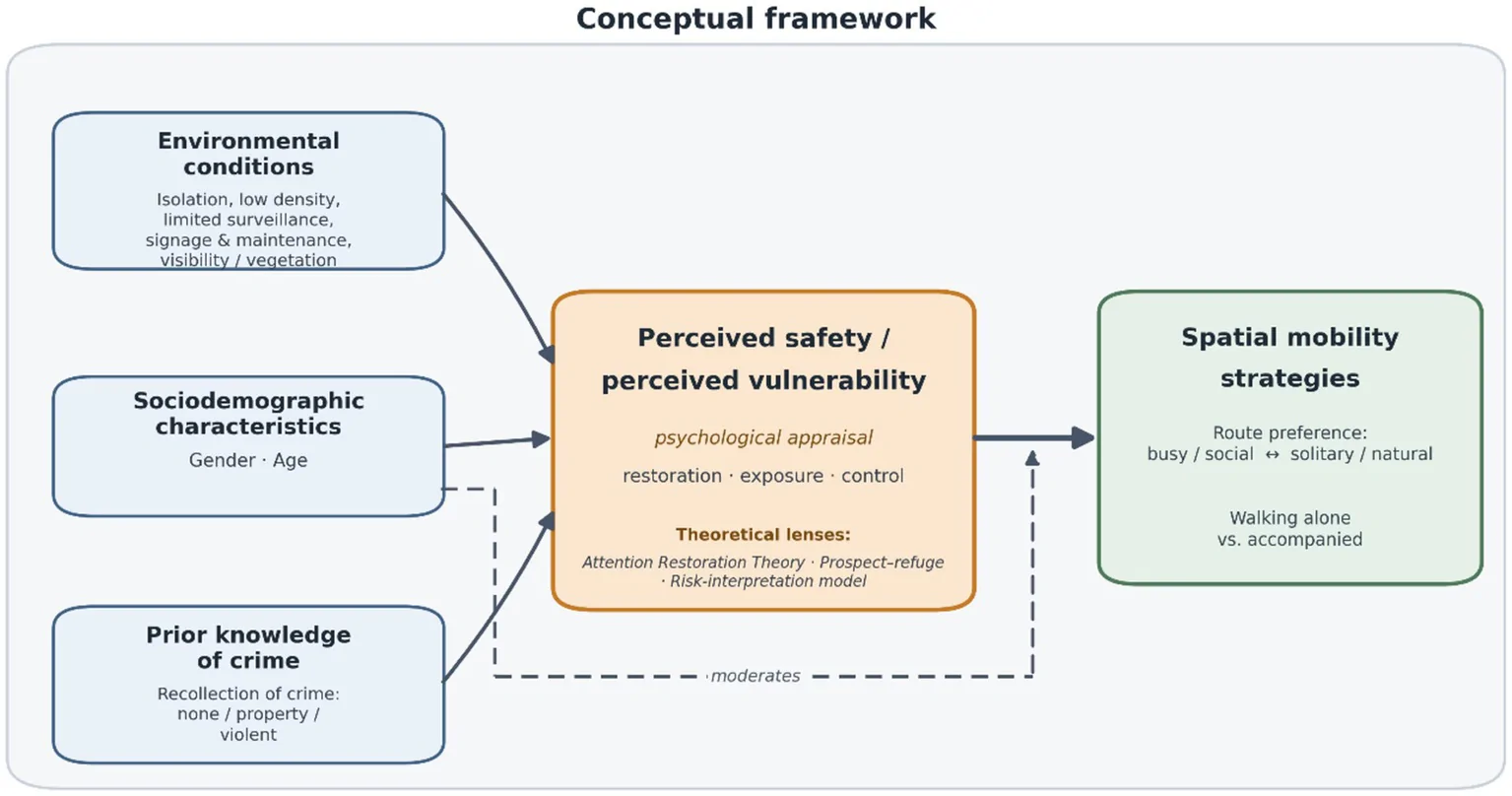
Conceptual framework of the study. Environmental conditions, sociodemographic characteristics and prior knowledge of crime are treated as antecedents of perceived safety/vulnerability, conceptualised as a psychological appraisal (restoration, exposure and control) informed by Attention Restoration Theory, Prospect–refuge theory and the Risk-interpretation model. This appraisal is, in turn, associated with pilgrims’ spatial mobility strategies (route preference and walking alone versus accompanied), with sociodemographic characteristics also acting as moderators. The figure depicts a conceptual model rather than an empirically tested path or mediation model.

By addressing these questions, the study aims to make three main contributions. Theoretically, it extends fear-of-crime research and environmental criminology beyond their traditional urban focus, showing how perceived risk structures spatial behaviour in large-scale natural and pilgrimage environments. Empirically, drawing on a sample of 1,080 pilgrims, it adopts a gender and age perspective that clarifies how perceived vulnerability differentiates the use of space across distinct sociodemographic groups. From an applied standpoint, the findings offer insights relevant to the design, management and safety of recreational and pilgrimage routes, informing interventions—consistent with the principles of Crime Prevention Through Environmental Design—intended to enhance perceived safety and to promote a more equitable and inclusive use of the Camino. In doing so, the study establishes a coherent link between the theoretical framework outlined above and the methodological, empirical and interpretive sections that follow (Figure 1).

## 2 Method

### 2.1 Participants

The sample consisted of 1,080 pilgrims (52.8% women, 47.2% men). In terms of age, 6.7% were between 18 and 29 years old, 20.5% between 30 and 44 years old, 47.3% between 45 and 59 years old, 24.3% between 60 and 75 years old. The remaining 1.3% were over 75 years old. In terms of educational attainment, 5.7% had primary education, 12.4% had secondary education, 26.9% had college level, 49.8% had a bachelor’s degree, and 5.2% had postgraduate studies. Statistical power for the sample was calculated using G*Power, version 3.1.9.7 (Faul et al., 2009) as a goodness-of-fit test for contingency tables, given an effect size *w* of 0.2 (small effect), an error probability *α* of 0.05, a sample size of 1,080, and 24 degrees of freedom (30 total categories minus 6 variables; Greenacre, 2007). Achieved power was estimated as 0.991, which can be considered very high.

### 2.2 Instruments

Scales measuring prior awareness of crime in the neighbourhood, comparative perceived safety, and perceived risk were used, developed *ad hoc* for this study, interspersing dichotomous responses with open-ended questions:

*Prior Knowledge of Crime in the Area*

- Are you aware of or have you heard of any serious crimes (rape, murder, etc.) have occurred on the Camino in the last 10 years? (*Yes/No*)
- If your answer was yes, what serious crimes do you know of that have occurred? Explain what you remember (*Open-ended question*)
- Are you aware of or have you heard of any misdemeanors such as robbery or theft that have occurred on the Camino in the last 10 years? (*Yes/No*)
- If your answer was yes, what minor offences or crimes do you know of that have occurred? Explain what you remember (*Open-ended question*)

*Comparative Perception of Safety:*

- Do you consider the routes of the Camino de Santiago to be safer than other similar routes? (*Yes/No*)
- Why? (*Open-ended question*)

*Perceived Risk/Vulnerability:*

- Would you walk the Camino de Santiago alone? (*Yes/No/Only some stages*)
- If your answer was no, why not?
- If your answer was only some stages of the Camino, indicate which one (*Urbanised area, rural/natural area/both*)
- Why? (*Open-ended question*)

*Preference for natural routes was also included as an ordinal* *variable:*

- How much do you enjoy walking in parks, on footpaths, or along nature trails in general? (*1-Minimal preference–5-Maximum preference*)

Additionally, the sociodemographic variables of *Gender* and *Age* were included to determine whether these variables, or any of them, could be influencing the development of protective and/or vulnerable behaviours.

### 2.3 Procedure

The questionnaire was conducted using the Google Forms platform and administered online through social media, reaching out to different pilgrim-related organisations and interest groups—including pilgrim associations and Jacobean confraternities (*asociaciones de amigos del Camino*), hospitaller (*albergue*) networks and online pilgrim communities and forums—so that it would reach as many pilgrims as possible. Participation was voluntary and anonymous, and respondents provided informed consent before completing the questionnaire.

## 3 Data analysis

All analyses in the study were performed using SPSS Statistics 29.0 (IBM Corporation, 2023). Educational level was not included in the study because preliminary analyses showed that it had no significant relationships with the other variables in the study. Six variables were finally included in the analyses; three of them were originally categorical: (1) *Gender*, with two categories (male and female); (2) *Age*, with five categories (18–29, 30–44, 45–59, 60–75, over 75); and (3) preference for natural routes (*Natural*), with five categories (1 = minimum preference; 5 = maximum preference).

The remaining three variables were obtained from the scales measuring prior awareness of crime, comparative perceived safety, and perceived risk. These scales included qualitative variables containing open-ended responses, so they were converted into categories based on an analysis of the content of the responses provided. To develop the categories, the terms most frequently used in the subjects’ responses were compiled, along with their frequency. Then reduced to a smaller number of more general categories, so that no category had a frequency of less than 10. Table 1 shows the categories of the study variables, along with their definitions. The resulting categorical variables were: (4) Recollection of crime (*Crime*), with 5 categories; this variable summarised all the questions related to prior knowledge of crime in the area. Those who did not remember any crime were assigned to one category (*No recall*); for those who did remember some crime, the type of crime was recorded using their qualitative responses (see Table 1). (5) Reasons for the perceived safety/insecurity (*Safety*), with 7 categories; this variable included all the questions related to comparative perception of safety. The reasons for perceived safety or unsafety were recorded using the qualitative responses (see Table 1). (6) Reasons for travelling alone or not (*Alone*), with 6 categories; this variable included all the questions related to perceived risk/vulnerability. The reasons for walking alone or not were recorded using the qualitative responses (see Table 1).

**Table 1:** Study variables, labels and associated categories, together with the definition of each category and the frequency for each category.

Variable (*Label*)	Categories (*Definition*)	N
Gender (*Gender*)	1. Male	510
	2. Female	570
Age (*Age*)	1. 18–29	72
	2. 30–44	221
	3. 45–59	511
	4. 60–75	262
	5. Over 75	14
Preference for natural routes (*Natural*)	1. Minimal preference	29
	2. Low preference	81
	3. Average preference	256
	4. High preference	302
	5. Maximum preference	412
Recollection of crime (*Crime*)	1. No recall (*Does not recall any*)	474
	2. Robbery/theft (*Robbery/theft of objects without violence*)	531
	3. Fraud/scam (*Deception without violence*)	10
	4. Assault/abuse (*Physical or verbal assault, abuse, rape*)	29
	5. Murder (*Homicide*)	36
Reasons for perceptions of safety or insecurity (*Safety*)	1. Presence of people (villages and *inhabited areas*)	40
	2. Companionship of fellow pilgrims (*Presence of other pilgrims*)	347
	3. Safer (*Perception of safety, no reason provided*)	232
	4. Not safer (*The Camino is no safer than other routes*)	187
	5. Signage/maintenance (Signage *and well-maintained surroundings*)	183
	6. Surveillance/assistance (*Presence of police, ambulances, etc.*)	41
	7. Lack of surveillance/assistance (*Opposite of the above*)	50
Reasons why I would or would not travel alone (*Alone*)	1. No reason (*Not specified*)	70
	2. I would travel alone (*I would travel alone, no reason provided*)	828
	3. Loneliness (*I would not go alone for fear of loneliness*)	82
	4. Fear/insecurity (*I would not go alone due to fear or insecurity*)	62
	5. Not alone (*I would not travel alone, no reason provided*)	25
	6. Accident/incident (*Would not go alone for fear of an accident, animal attack, problem, etc.*)	13

In a second step, bivariate measures of association between the study variables were calculated. Given that most of the study variables were polytomous, the chi-square based contingency coefficient was calculated as a measure of association.

Finally, all study variables were analysed together using multiple correspondence analysis (Gifi, 1990; Greenacre, 2007). Multiple correspondence analysis (MCA) allows a large set of categorical variables to be represented in a smaller set of orthogonal components or dimensions. The analysis assigns quantifications in these dimensions simultaneously to both the categories of the variables and the cases (objects), so the same criteria can be used to interpret their position in the space: similar categories (or objects) will be closer in the space defined by the dimensions, while dissimilar ones will be far apart in that same space. Since both categories and objects are represented in the same space, the interpretation of the dimensions of that space will be the same in both cases. Solutions were requested in 3 and 2 dimensions, using the symmetric normalisation method in both cases, which is more useful when comparing the results of both categories and objects.

## 4 Results

### 4.1 Preliminary results

Table 2 shows the bivariate relationships between the study variables, according to their contingency coefficient values, together with their significance. Although some relationships were highly significant, values for the corresponding coefficients were moderate in general.

**Table 2:** Contingency coefficient values for the study variables, together with their significance.

Variable	Gender	Age	Crime	Natural	Safety
Age	0.241***				
Crime	0.137***	0.173**			
Natural	0.122**	0.147	0.140		
Safety	0.109*	0.174	0.244***	0.182	
Alone	0.325***	0.173	0.198***	0.263***	0.209*

*p < 0.05; **p < 0.01; ***p < 0.001.

The results in Table 2 show that gender was significantly related to all variables in the study. Inspection of the observed and expected frequencies for the corresponding tables showed a higher-than-expected frequency of women at younger ages, a trend that reverses after age 60, when there is a higher-than-expected frequency of men. In terms of recollection of crimes, women tend to indicate that they do not remember any. About preference for natural routes, men show higher than expected frequencies for natural routes, while women show the opposite trend. As for the reasons for perceiving safety or not, women tend to indicate that they find the route safe, while men generally do not rate it as safer than other routes. Finally, regarding the reasons for travelling alone or not, men indicate more frequently than expected that they would travel alone, while for women the opposite is true, due to reasons such as loneliness, fear/insecurity, or accidents/incidents.

Apart from *Gender*, *Age* also showed significant relationships with recollection of crimes and with reasons for travelling alone or not. In the case of recollection of crimes, younger subjects (18–29 years old) tend not to remember any crimes to a greater extent than the rest. Regarding the reasons for travelling alone or not, there are fewer subjects than expected who would travel alone in the younger age group (18–29 years old).

The recollection of crimes showed significant relationships with all variables except for the preference for natural routes. Regarding the reasons for the perceived safety or not, subjects who perceive the route as safer tend not to remember any crimes, while those who do not perceive it as safer tend to remember robbery/theft more often than expected. With respect to the reasons why they would or would not go alone, subjects who would go alone tend to mention robbery/theft more than expected, while those who would not go alone tend not to recall any crime.

In addition to gender, preference for natural routes showed a significant relationship with reasons for safety or insecurity and with reasons for travelling alone or not. In the first case, subjects more favourable to natural routes tend to show less interest in the presence of people or other pilgrims. In the second case, subjects who were more favourable to natural routes tended to indicate that they would go alone more often than expected, while those who were less favourable showed the opposite tendency.

Finally, the reasons for safety or insecurity showed significant relationships with all other variables. In the case of their relationship with the reasons for travelling alone or not, those who would travel alone tend to indicate that they consider the route to be safe, while among those who would not travel alone, the lack of surveillance/assistance is cited as a reason for insecurity more often than expected.

### 4.2 Multiple correspondence analysis

The aim for the analysis was to obtain the most parsimonious model possible that adequately represents the information provided by all the variables. The comparison between the two-dimensional and three-dimensional solutions showed that the two-dimensional solution was the most appropriate, given that the third dimension did not contribute to the interpretability of the result and its contribution, both in terms of inertia and in terms of discriminant measures, was small.

With respect to the two-dimensional solution, the first dimension was slightly more important (Cronbach’s alpha = 0.486; eigenvalue = 1.68; % variance = 28.01%) than the second (Cronbach’s alpha = 0.304; eigenvalue = 1.34; % variance = 22.33%). Table 3 shows the discriminant measures for the study variables for each of the dimensions of the solution.

**Table 3:** Discriminant measures of each dimension (contributions to total eigenvalue) for the study variables.

Variable	Dimension 1	Dimension 2
*Age*	0.174	**0.283**
*Crime*	**0.233**	0.159
Preference for natural routes (*Natural*)	0.235	0.225
Reason for safety/unsafety (*Safety*)	0.077	**0.380**
Reason for going alone or not going alone (*Alone*)	**0.499**	0.199
*Gender*	**0.463**	0.096
Total active (eigenvalue):	1.681	1.340

Bold values indicate the dimension on which each variable shows its highest discriminant measure.

The variables most closely related to dimension 1 are *Gender* (0.463) and *Alone* (0.499), while the variable most closely related to dimension 2 is *Safety* (0.380). The rest of the variables have weights that are more evenly distributed between both dimensions, although *Age* tends to be slightly more related to dimension 2 (0.283). *Crime* is somewhat more related to dimension 1 (0.233). For its part, *Natural* tends to be related almost equally to both dimensions (0.235 and 0.225).

**Figure 2:**
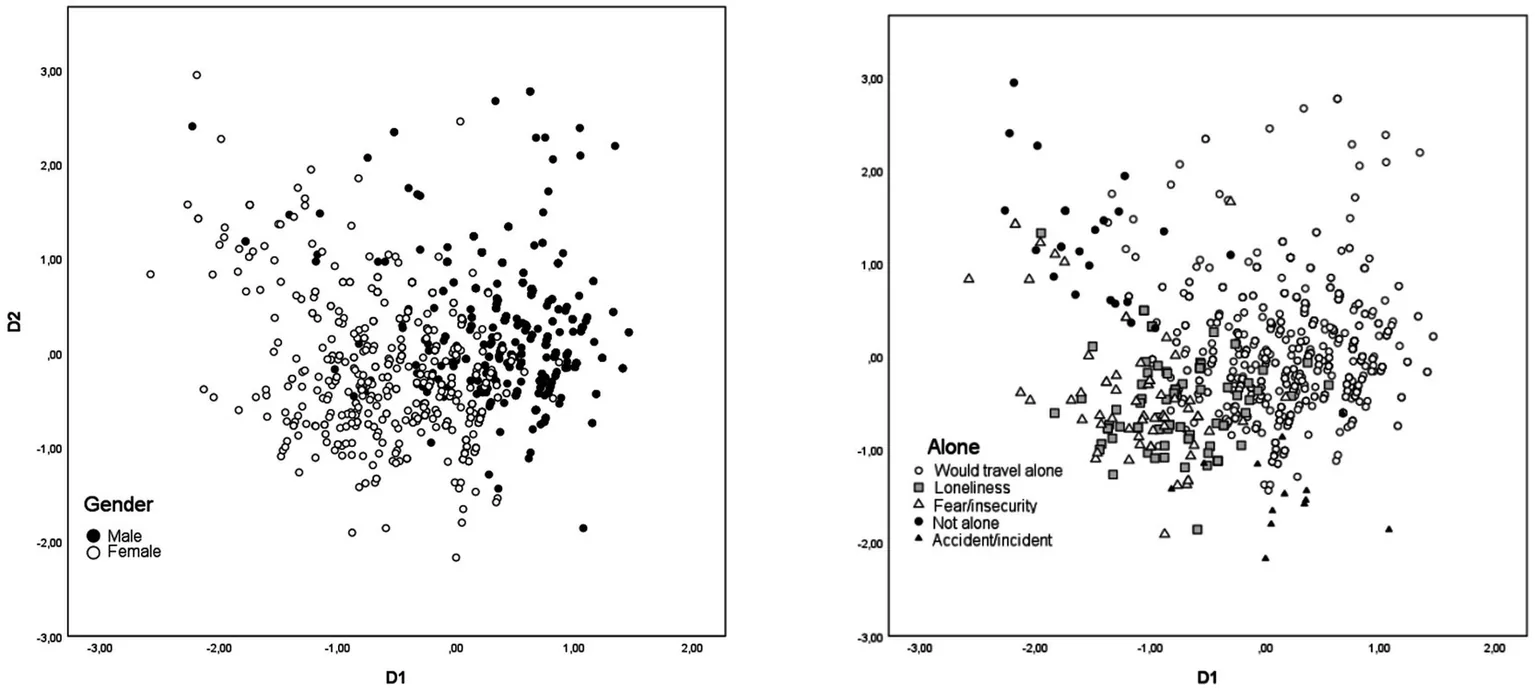
Representation of cases (objects) in the two-dimensional space of the MCA solution, according to gender and reasons for travelling alone or not (*Alone*).

Figure 2 shows the representation of the cloud of cases (objects) in the two-dimensional solution as a function of the variables most related to dimension 1 (horizontal) of the MCA: *Gender* and *Alone*. This dimension clearly discriminates between women, located predominantly on the left, and men, located predominantly on the right. At the same time, it can also be seen that those who prefer to travel alone (located on the right) are predominantly men. Those who would not travel alone are grouped on the left. Of these, those who do not report any reason are in the upper left, while those who report reasons such as loneliness or fear are in the centre and left, and those who fear the possibility of an accident/incident tend to be in the lower centre. This result indicates a lower inclination among women to travel alone and coincides with the interpretation of the relationship between the two variables based on Table 2. Thus, dimension 1 seems to discriminate between those subjects who do not mind travelling alone (generally men), located on the right, and those who prefer to travel accompanied (generally women), located on the left.

Dimension 2 is more easily interpreted from the positions of the variable categories in the two-dimensional space provided by the MCA (see Figure 2). Regarding the variable most closely related to this dimension (*Safety*), subjects who consider the routes to be safe are in the upper part of the graph, while the rest of the categories are in the lower part. In the case of age, which is also related to this dimension, older subjects (aged 60 and over) are in the upper zone, and those under 60 in the lower zone.

**Figure 3:**
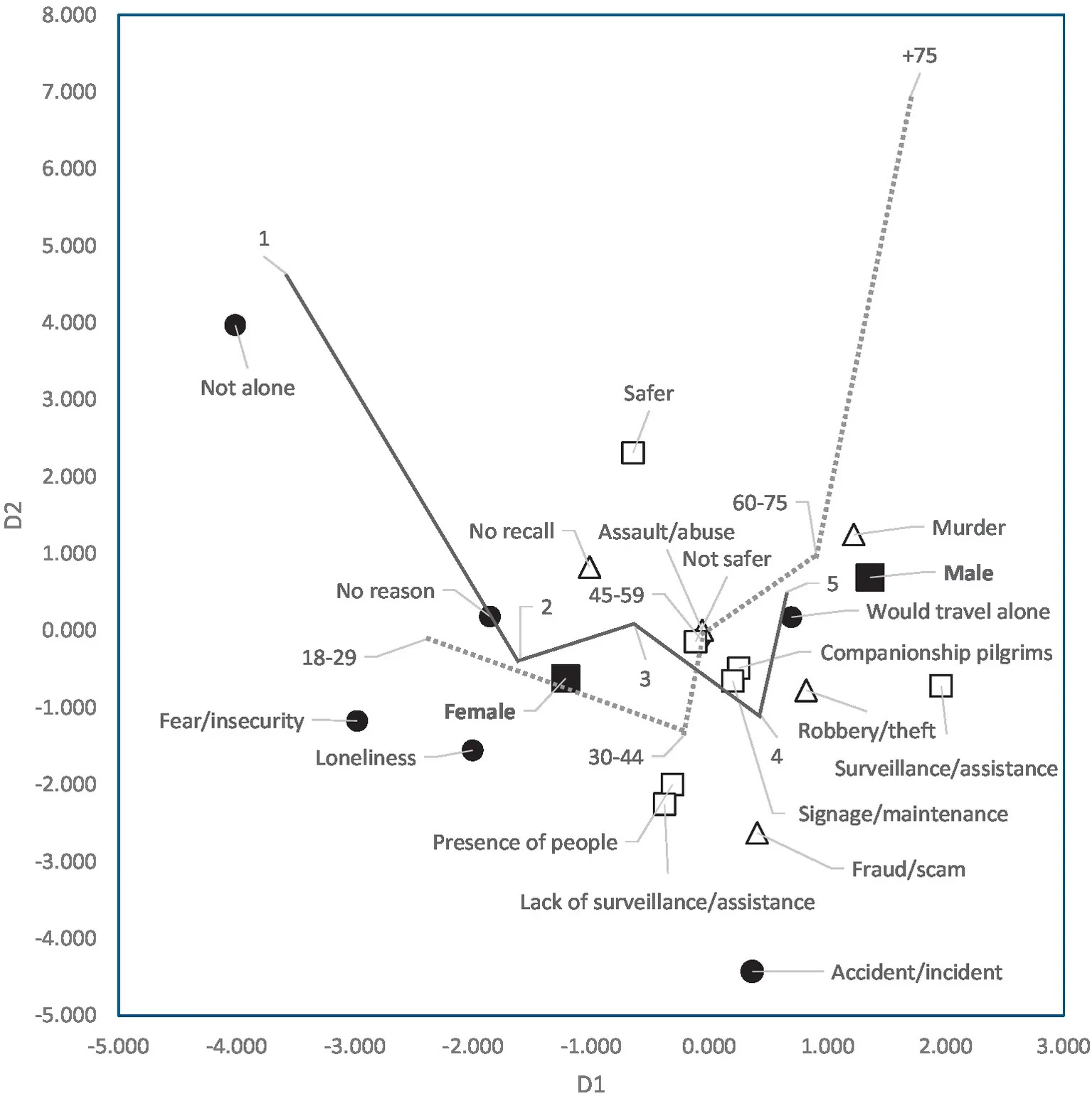
Dimensions 1 and 2 of the multiple correspondence analysis solution and categories of the variables included. Keys: ■ Gender; -- Age; Δ Crime; __ Natural; ● Safety; □ Alone.

Figure 3 confirms the relationship between *Gender* and *Alone* with dimension 1: the categories corresponding to men and travelling alone are located on the right, while those corresponding to women and not travelling alone are located on the left. In terms of remembering crimes, subjects who do not remember are located on the left, while those who do remember are located on the right.

Finally, interest in nature trails is lower on the left (categories 1, 2 and 3) and higher on the right of the graph (categories 4 and 5). This is probably because the more natural trails are also more solitary, and subjects on the left prefer not to go alone.

Thus, the solution provided by the MCA identifies two dimensions underlying the study variables. The first dimension relates to a preference for natural routes, travelling alone, being older and being male, on the one hand, and a preference for travelling with others and on busy routes with other people around, due to fear, being a young or middle-aged woman, on the other. Dimension 2, meanwhile, distinguishes between subjects who consider the route to be safe, regardless of whether they prefer busy routes and company (upper left area), or do not mind going through less busy areas or alone (upper right area). In the lower area are those who do not feel safe, either because of the possibility of accidents or unpleasant encounters, or because of the lack of surveillance or assistance. For these subjects, the presence of people or surveillance alleviates their fear, insecurity and loneliness.

Finally, it should be noted that, due to the characteristics of the MCA, those characteristics with lower frequencies (i.e., with lower mass) tend to be located at more extreme positions in both dimensions, while those with higher frequencies tend to be in the centre, due to their greater mass. This is why those over 75 years of age (*N* = 14) appear in extreme positions, those who would not go alone (*N* = 25); those who have a lower preference for natural routes (*N* = 29); those who remember crimes such as fraud or scams (*N* = 10); or those who are concerned about the possibility of having an accident or a bad encounter (*N* = 13) appear in extreme positions. Thus, the extreme position of a given category would indicate its exceptionality or rarity, as it corresponds to a small group of subjects in the sample.

## 5 Discussion

The results of this study confirm that perceived safety on the Camino de Santiago is a complex psychosocial phenomenon resulting from the interaction between individual, sociodemographic and environmental factors. Rather than responding exclusively to objective indicators of crime or the actual occurrence of victimisation, perceptions of safety emerge as a subjective construct that appears to shape how individuals interpret their surroundings and organise their spatial behaviour. This finding is consistent with a large body of research on fear of crime, which has shown that individuals often adjust their daily practices according to perceived vulnerability rather than actual risk levels (Ferraro, 1995; Hale, 1996; Jackson, 2005).

The results also highlight the importance of integrating a gender perspective into the planning of the Camino. The differences observed between men and women in their perception of risk and mobility strategies show that experiences of space are not homogeneous (Foster et al., 2004). Rišová and Sládeková Madajová (2020) likewise report that young and middle-aged women more often perceive insecurity and fear of assault or harassment, especially in low-density, poorly lit areas, areas frequented by strangers, and poorly monitored streets. Designing routes and services that take these differences into account contributes not only to improving the perception of safety among the most vulnerable groups, but also to promoting a more equitable and inclusive use of the Camino. In this regard, measures aimed at reducing the feeling of isolation, facilitating informal accompaniment, or reinforcing the visibility of other users can be particularly effective. Pérez-Tejera et al. (2022), for example, found that spaces with greater perceived safety in public parks attracted more women, heterogeneous groups, people of different ages, and people accompanied by others, suggesting that the presence of other people and social grouping function as a factor of comfort and safety, especially for women.

Importantly, these results reinforce the idea that natural environments are not intrinsically restorative, but rather that their beneficial potential depends on their being perceived as safe and manageable spaces, as already observed in a study by Subiza et al. (2020). When the natural environment is interpreted as excessively isolated, lacking institutional support or difficult to control, the psychological benefits associated with contact with nature—such as stress reduction, attentional recovery or improved well-being—can be significantly limited. In the case of the Camino de Santiago, this issue takes on particular relevance, given that it is a prolonged experience involving continuous travel, exposure to changing environments, and constant decisions about routes and modes of transport.

Beyond confirming that natural settings can support well-being, these findings qualify a central assumption of the restorative-environments literature. Whereas research on restoration has tended to treat exposure to nature as broadly beneficial (Kaplan and Kaplan, 1989; Groeneveld et al., 2025; Matias et al., 2022), the present results indicate that the restorative potential of large-scale natural environments is conditional on—and unequally distributed by—perceived safety. Because perceived safety is itself structured by gender and age, access to the psychological benefits of the Camino is socially stratified: women and younger pilgrims, who report greater perceived vulnerability, more often confine their mobility to socially populated routes, whereas men and older pilgrims move more freely through solitary, highly natural segments. This extends the work of Subiza et al. (2020) and Pérez-Tejera et al. (2022) from bounded urban green and blue spaces to a prolonged, mobile and linear setting, suggesting that perceived safety operates not as a single appraisal of a fixed site but as a continuous regulatory process shaping restorative opportunity across an extended journey.

From the perspective of environmental criminology, the interaction between environmental cues and individual characteristics plays a crucial role in shaping perceptions of safety and spatial decision-making. Research in this field has highlighted how the design and organisation of space influence both opportunities for crime and perceptions of risk (Brantingham and Brantingham, 1991; Clarke, 1997; Felson, 2002). Consistent with this perspective, the present study shows that pilgrims’ mobility strategies are structured around subjective assessments of safety that influence route selection, preferences for solitary or busy environments and decisions about travelling alone or accompanied.

From the standpoint of environmental criminology, the contribution is twofold. First, the study relocates fear-of-crime and CPTED reasoning from the urban micro-settings in which they were developed (Brantingham and Brantingham, 1991; Cozens and Love, 2015) to a large-scale linear natural route in which objective victimisation is rare, showing that perceived risk can structure spatial behaviour even in the near-absence of crime. Second, it reframes the notion of guardianship: in routine-activity and CPTED accounts, natural surveillance is typically supplied by residents, passers-by or formal devices anchored to a fixed built environment (Felson, 2002), whereas on the Camino the relevant guardians are co-present yet itinerant strangers—other pilgrims—so that informal surveillance becomes mobile and transient. The strong association between a preference for busy routes and reluctance to walk alone, particularly among women, suggests that social presence functions as a portable form of guardianship that frameworks designed for static settings do not fully capture.

The multiple correspondence analysis identified two main dimensions underlying these behavioural patterns. The first dimension reflects differences associated primarily with gender and the willingness to walk alone. Women and younger pilgrims tend to prefer routes where the presence of other people is more likely, while men and older participants are more inclined to choose more solitary or highly natural routes and to travel alone. These patterns echo previous research showing that fear of crime and perceived vulnerability vary significantly across sociodemographic groups, with women often reporting higher levels of perceived risk in public spaces (Pain, 2001; Stanko, 1990).

The second dimension identified in the analysis relates more directly to the perception of safety itself. Participants who consider the Camino to be a generally safe environment appear in a distinct cluster regardless of whether they prefer busy routes or solitary ones. In contrast, those who perceive greater insecurity tend to emphasise concerns related to the lack of surveillance, the possibility of accidents or the absence of assistance in remote areas. These findings accord with studies in environmental criminology showing that environmental characteristics such as visibility, accessibility and informal guardianship influence perceptions of safety and risk (Fisher and Nasar, 1992; Cozens and Love, 2015).

Although most fear-of-crime research has focused on urban environments, the present findings suggest that similar mechanisms operate in large-scale natural settings. Characteristics commonly associated with natural landscapes—such as isolation, limited surveillance and low population density—may increase perceived vulnerability among certain users, particularly those who already perceive themselves as more exposed to potential victimisation. Recent research has also shown that environmental features and spatial configuration play a key role in shaping perceptions of safety and risk in public spaces (Kuen et al., 2022; Wen et al., 2024). The present study extends these insights to pilgrimage routes and natural environments, highlighting how perceived risk influences mobility strategies even in contexts where actual crime levels may be relatively low.

A further, more critical observation concerns prior knowledge of crime, which did not operate in the linear fashion often assumed in media-and-fear research (Focás, 2015). Pilgrims willing to walk alone recalled property crime more frequently than expected, whereas those who regarded the route as safe tended to recall no crime at all. Rather than uniformly heightening precaution, awareness of crime appears to be filtered through appraisals of personal control and coping, in line with Ferraro’s (1995) risk-interpretation model but extending it to a recreational natural context. Information about crime and fear of crime thus appear only loosely coupled, implying that interventions premised on a direct information–fear pathway may be misdirected.

More broadly, the findings highlight the importance of considering perceived safety as a key factor influencing the use of natural recreational environments. Environmental criminology has long emphasised the role of spatial design and environmental management in shaping perceptions of safety and opportunities for crime (Brantingham and Brantingham, 1991; Clarke, 1997). Approaches such as Crime Prevention Through Environmental Design (CPTED) underline the importance of factors such as visibility, maintenance, territoriality and natural surveillance in promoting safer environments (Cozens and Love, 2015; Cozens et al., 2023). In the context of pilgrimage routes such as the Camino de Santiago, these principles suggest that maintaining visible signage, ensuring adequate maintenance of paths and providing accessible information about assistance services may contribute to reinforcing pilgrims’ perceptions of safety.

The Camino de Santiago therefore offers a particularly valuable setting for analysing how perceived risk shapes spatial behaviour in natural environments. Unlike most urban contexts commonly studied in criminology, pilgrimage routes involve extended journeys through heterogeneous landscapes where individuals must continuously evaluate environmental cues and adapt their behaviour accordingly. Understanding these dynamics is essential for developing strategies that promote both safety and accessibility in recreational routes and long-distance trails.

### 5.1 Limitations

These contributions should be read in light of several limitations. The data are cross-sectional and self-reported, drawn from a self-selected online sample that over-represents older and university-educated pilgrims; the associations identified are therefore correlational and may not generalise to the wider pilgrim population. No objective indicators of victimisation were available, precluding a direct comparison between perceived and actual risk. The preference for solitary, highly natural routes may also reflect a deliberate search for spiritual solitude rather than lower fear, so that motivation and perceived safety are probably entangled in ways the present design cannot disentangle. Finally, as is inherent to multiple correspondence analysis, low-frequency categories occupy extreme positions and warrant cautious interpretation. Longitudinal and mixed-method designs combining perceived-safety measures with objective route characteristics would help to clarify the direction of these relationships.

## 6 Conclusion

The results indicate that perceptions of safety are closely associated with sociodemographic characteristics, environmental features and spatial mobility strategies, and appear to operate as an intervening appraisal between them rather than as a formally tested mediator. Gender, age, knowledge of crime and environmental perceptions interact to produce differentiated patterns of route selection and travel behaviour. By extending fear-of-crime research to large-scale natural environments, this study contributes to a growing body of work exploring how perceived risk shapes the use of public and recreational spaces. More specifically, by treating perceived safety as a precondition for environmental restoration (environmental psychology) and as a mobile, socially supplied form of guardianship (environmental criminology), the study connects two literatures that have largely developed in parallel. Future research should continue to examine these dynamics through longitudinal and mixed-method approaches, allowing a deeper understanding of how perceptions of safety evolve over time and how specific environmental interventions may influence spatial behaviour.

## Data Availability

The raw data supporting the conclusions of this article will be made available by the authors, without undue reservation.
